# Buschke - Loewenstein tumor resection with simultaneous reconstruction of extensive tissue losses: case report

**DOI:** 10.1186/s12893-015-0026-0

**Published:** 2015-04-10

**Authors:** Urszula Skowrońska-Piekarska, Tomasz Kościński

**Affiliations:** The Chair and Department of General, Endocrine and Gastroenterological Oncology Surgery, Karol Marcinkowski University of Medical Sciences in Poznań, ul. Przybyszewskiego 49, 60-355 Poznań, Poland

**Keywords:** Buschke, Loewenstein tumor, surgical intervention, simultaneous loss reconstruction

## Abstract

**Background:**

Giant condyloma acuminatum or Buschke - Loewenstein tumor is a very rare disease usually located in the genital, anorectal, and perianal regions. It is locally invasive but in mostly cases displays a benign cytology on preoperative tissue sampling. Because of its low incidence little is known about treatment outcomes. Complete surgical excision is the treatment of choice. Different surgical methods have been applied to reach curability. To our knowledge such an advanced sized tumors in this localization has only been reported few times before with different surgical techniques being applied.

**Case presentation:**

We describe a case of 56 years old female with 20 years persisting condyloma acuminatum progressing to a very huge dimensions perianal Buschke-Lowenstein tumor with one of the widest excision in the literature without the need for diverting stoma. The tumor size and its location determined the choice of treatment option and suspected prognosis for the patient outcome. Treatment was impeded by patient’s malnutrition. The giant Buschke - Loewenstein tumor was resected from the anus, perineum and gluteal areas. The large tissue losses were simultaneously covered with rotational skin and fatty subcutaneous tissue flaps, mobilized from neighboring gluteal and femoral areas. The circumferential part of the anal canal was covered with skin grafted from the mentioned flaps and it was attached to the anal mucosa. No protective stoma was formed. Despite temporary problems with healing of the covering skin flaps, full permanent coverage of the resection site has been achieved. Anal canal function has also improved within the time.

**Conclusion:**

The patient with BLT must be very carefully clinical and imagistic investigated in order to detect the tumor visceral invasion and to establish the extension of the surgical procedure. There exists an extensive and time-consuming surgical procedure which allows to remove the giant anorectal Buschke - Loewenstein tumors with good function of the anorectum and without the necessity of diverting stoma creation.

## Background

Condyloma acuminatum (CA) caused by Human Papillomavirus (HPV) is the infection in the anogenital region mostly occurring as a sexually transmitted disease. The local activity of HPV expresses itself in the overgrowth of the epithelium of the affected tissues [1-3]. The prevalence of human papilloma virus is 10% in the world, mostly it affects women under the age of 35. The associated CA has the prevalence of 0.75–3% in Europe and 1% in the United States [3]. One of the risk factors for CA development is an immune-compromised state [3]. The progression of CA to a Buschke-Lowenstein tumor (BLT) occurs on very rare occasions. BLT known also as giant CA is a slowly growing cauliflower-like tumor associated with HPV types 6, 11, 16, and 18, but unlike simple condyloma, it is locally destructive and infiltrative. Due to its very low incidence rate, mainly sporadic single center experience is available in literature [3,4]. It is reported in the current literature that BLT incidence increased to 6.3 cases per year in the last decade worldwide [3]. Giant condyloma acuminatum presents with a 2.7:1 male-to-female ratio, the mean age at presentation is 43.9 years. The most common presenting symptoms are perianal mass (47 percent), pain (32 percent), perianal abscess or fistula (32 percent), and bleeding (18 percent) [5]. The disease, for which the most important treatment method is the surgical excision, differs from normal condyloma acuminatum cases with its high degree of malignancy. In 40-60% of condyloma acuminatum cases malignant transformation into invasive squamous carcinoma in particular for HPV types 16 and 18 is proved [1-3]. Foci of invasive carcinoma are noted in 50 percent of the reports, “carcinoma in situ” in 8 percent, and no invasion in 42 percent [5]. Local invasion and local recurrence are the major source of morbidity in this disease. The disease is associated with high recurrence and mortality rates of, respectively, 67 and 21 percent [3,4,6]. It is why complete surgical excision is the treatment of choice and often wide wounds are necessary to reach clear margins and prevent recurrence [1,4]. However widespread anodermal and epithelial condylomata excision rises the risk of scar formation and possible anal stenosis. But as a primarily applied treatment surgical excision has the highest success rate and the lowest risk of recurrence [3,6]. In limited lesions primary excision can be safely performed leaving wounds open to granulate while in more extensive lesions flap or skin graft coverage according to different techniques is preferable to decrease the length of recovery and minimize risk of severe anal stricture [4]. Abdominoperineal resection should be performed for more extensive lesions with deep anorectal invasion, malignant transformation or malignant tumor recurrence. Recurrence of BLT is common. No sufficient data are available to recommend any medical treatment such as interferon, radiotherapy or chemotherapy, with all their limitations and side or adverse effects [3,7].

The authors present a case of a very extensive BLT treated with an S-plasty rotating and a bilateral house advancement flap with Burow’s triangles cuts respectively with good functional result.

## Case presentation

A 56 years old female, chain-smoker, admitted to the Department on February 15, 2012 for a giant tumor in the perianal area, covering the whole perianal and gluteal regions, complicated with persistent bleeding and discharging. Blood loss caused severe anemia, which required multiple transfusions of haematogenous/blood replacement products.

The tumor had been growing in the patient for almost 20 years. Due to feelings of shame with her condition she did not seek any medical advice until the overgrown tumor has started to give her problems with acquiring a sitting position. Disintegrating tumor tissue was producing a discharge and an unpleasant odor, easily smelled by the patient and people around her. The tumor also caused problems with defecation, constipation and incontinence of gases and stool, which lead to decreased food intake and progressing loss of body mass.

**Figure 1:**
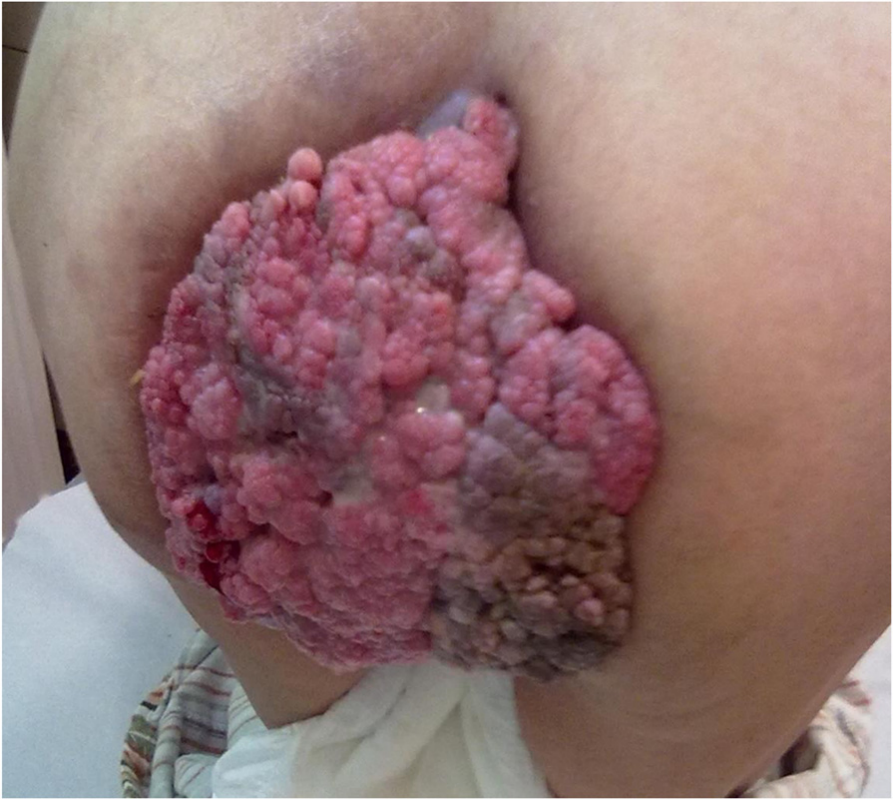
**Extensive Buschke-Loewenstein tumor of the perianal region.**

**Figure 2:**
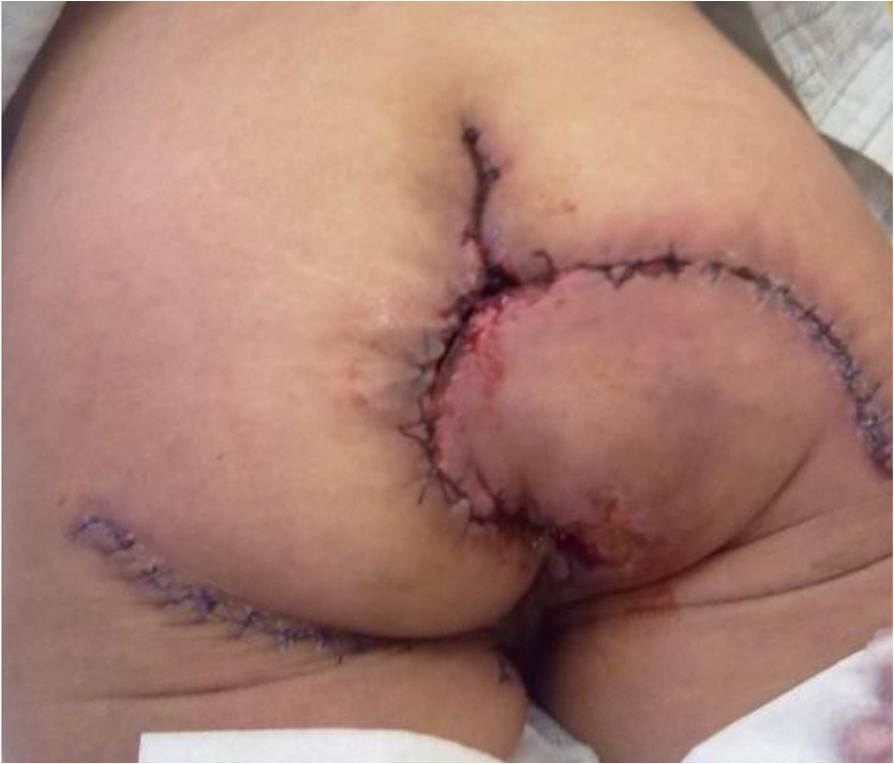
**The healing process in the grafted skin and subcutaneous tissue flaps.**

**Figure 3:**
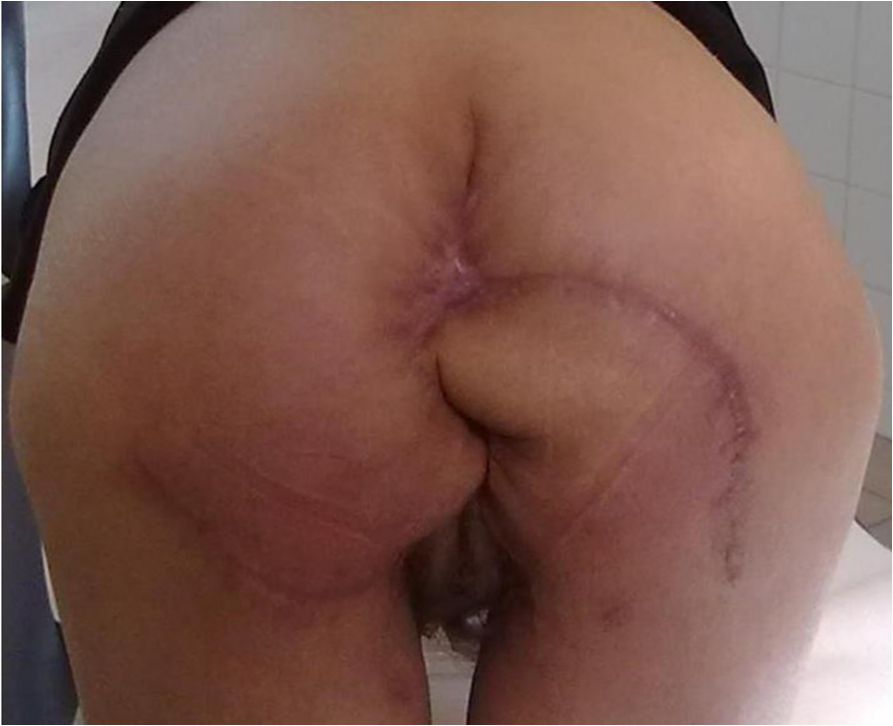
**The full wound healing process at 6 months follow-up.**

**Figure 4:**
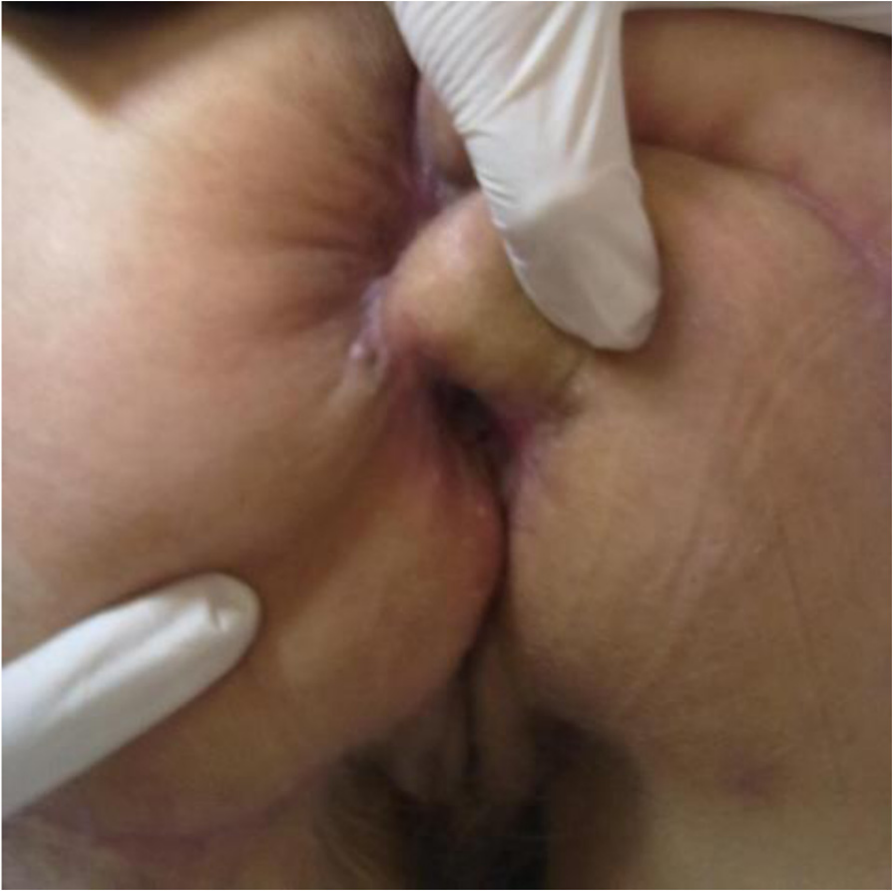
**The scar stenosis at the skin border of the anal canal which was treated with anal dilatator.**

At the date of admission a giant cauliflower like tumor was reported in the perianal area. The tumor was 18 cm x 20 cm in diameters, and was closing the anus almost completely. It was invading the lower anal canal, skin in the crotch area and at the buttocks. The tumor was bleeding and was covered with disintegrating tissue with areas of localized necrosis (Figure 1). Viral serology revealed the patient to be HPV-16 and HPV-18 negative. Her HIV status was negative. Laboratory tests showed anemia and also confirmed a high level of malnutrition. Pathologic tests of the multiple biopsies of the tumor showed condyloma acuminatum. Imaging tests (abdominal USG, abdominal and pelvic MRI, X-ray of the chest) excluded distant metastases, as the tumor was suspected to be malignant, and proved that the mass did not invade anal sphincters but was compromised to anal mucosa. On February 22^nd^, 2012 a full tumor resection was performed together with the removal of subcutaneous tissue with the anoderm of the whole anal canal circuit. The weight of the tumor immediately after removal was 1.650 kg. The tissue loss was covered with two rotational skin and fatty subcutaneous tissue flaps in butterfly wing shapes, which were grafted from neighboring areas of buttocks and thighs. The whole circuit of the lower anal canal was covered with skin taken from those lobes and an anastomosis with the anal mucosa was performed. To align the length of the wound edges and to reduce the tension after moving the lobes, Burow’s triangles were cut at the base of the lobes. Two Redon’s drains were left in the wound and no protective stoma was formed. Postoperative recovery was complicated with temporary ischemia of the edge of one of the grafted lobes. After implanting secondary stitches, full coverage of the wound was achieved (Figure 2). In the pre- and postoperative periods, a full, complete parenteral nutrition was applied, this also helped to delay the first postoperative defecation. Patient was discharged home on day 32, before the wound was fully healed. The 2 and 6 months follow-up examinations showed full wound healing and a normal activity of the anus. The stenosis of the anus along the stitch lines were expanded using the dilatator (Figures 3 and 4). After surgery the patient’s subjective quality of life improved a lot, mainly due to the full, radical tumor removal with its odor, restored ability to function normally including sitting and regular defecation, restored ability to function among people, and because no stoma had to be performed. Postoperative pathologic tests showed huge genital warts with characteristic of a Buschke-Loewenstein tumor. In the 12 months follow-up examination a 2.5 over 2.7 cm recurrent tumor was found in the anal canal, and it was removed without compromising the function of the anus. The 18, 24 and 30 months follow-up examinations didn’t reveal any local recurrence signs nor any other pathologies.

## Conclusions

Although the incidence of the BLTs appears to be increasing, for patients with this rare condition there is still a lack of evidence on the appropriate methods of treatment. Early detection of the BLT, which mostly depends on patient’s wish, with a reliable pathological diagnosis is essential for applying the appropriate treatment and predicting treatment outcome. The detection or exclusion of malignant transformation in the giant tumors can be very difficult to obtain. It is well accepted that there is no constant histological characteristic of BLT which rather exhibit a continuum between condyloma acuminatum and squamous cell carcinoma [8]. The large BLTs cause possible sampling errors. To some extend patient factors, like their nutrition or immune state disorders, may determine the choice of treatment [4,5,7]. The cell-mediated immune response is essential in the natural control of HPV infections [2,7]. The patient with BLT must be very carefully clinically and imagistic (CT or MRI) investigated in order to detect the tumor visceral invasion. The prompt and adequate treatment of a BLT is needed to cut down the risk of local invasiveness which may lead to various complications such as abscess formation, fistulas, and defecation problems. [1,3,9] The method of choice is surgery with wide perianal excision with pathological margins control. The tumor still has a high recurrence rate of 50% in spite of radical or very wide local excision [2,8,10]. The radical pelvic surgery is indicated only in patients with proved visceral invasion. The efficacy of chemo and/or radiotherapy is not satisfying so far [1-3,7] The administration of interferon is still experimental treatment and because of its potential life-threatening risks should be considered as a last instance option [3,5,8].

Our case of great size BLT involving bilaterally gluteal and perineal skin, anal and vulva’s mucosa with no malignant transformation was treated by wide tissue excision. Very large skin and mucosa loses were subsequently covered by two rotational skin and fatty subcutaneous tissue flaps. Thus the anal function has been preserved. Total parenteral nutrition postponing defecation enabled avoiding a diverting stoma creation. This suggests that a stoma can be safely avoided in selected patients, and fits in with the observation that in up to 70% of patients a stoma can be omitted, with function recovery and no complications after wide perineal and perianal excisions with flap procedures [11]. After a small recurrent anal tumor removal the patient is free of the disease for 30 months now.

### Consent

Written informed consent was obtained from the patient for publication of this Case report and any accompanying images. A copy of the written consent is available for review by the Editor of this journal.

## Abbreviations

CA: condyloma acuminatum
HPV: Human Papillomavirus
BLT: Buschke-Loewenstein tumor

## References

[1] Tas S, Arik MK, Ozkul F, Cikman O, Akgun Y. Perianal giant condyloma acuminatum-buschke-löwenstein tumor: a case report. Case Rep Surg*.* 2012. http://dx.doi.org/10.1155/2012/507374. 10.1155/2012/507374 PMC3508531 23213594

[2] Papiu HS, Dumnici A, Olariu T, Onita M, Hornung E, Goldis D (2011). Perianal giant condyloma acuminatum (Buschke-Löwenstein tumor). Case report and review of the literature. Chirurgia (Bucur).

[3] Wester NE, Hutten EM, Krikke C, Pol RA. Intra-abdominal localisation of a buschke-lowenstein tumour: case presentation and review of the literature. Case Rep Transplant. 2013. http://dx.doi.org/10.1155/2013/187682. 10.1155/2013/187682 PMC3789409 24159412

[4] Mingolla GP, Potì O, Carbotta G, Marra C, Borgia G, De Giorgi D (2013). Reconstructive surgery in anal giant condyloma: report of two cases. Int J Surg Case Rep.

[5] Trombetta LJ, Place RJ (2001). Giant condyloma acuminatum of the anorectum: trends in epidemiology and management: report of a case and review of the literature. Dis Colon Rectum.

[6] Chu QD, Vezeridis MP, Libbey NP, Wanebo HJ (1994). Giant condyloma acuminatum (Buschke-Lowenstein tumor) of the anorectal and perianal regions: analysis of 42 cases. Dis Colon Rectum.

[7] Haque W, Kelly E, Dhingra S, Carpenter LS (2010). Successful treatment of recurrent buschke-Lowenstein tumor by radiation therapy and chemotherapy. Int J Colorec Dis.

[8] Gholam P, Enk A, Hartschuh W (2009). Successful surgical management of giant condyloma acuminatum (Buschke-Löwenstein tumor) in the genitoanal region: a case report and evaluation of current therapies. Dermatology.

[9] Spinu D, Rǎdulescu A, Bratu O, Checheritǎ IA, Ranetti AE, Mischianu D (2014). Giant condyloma acuminatum – Buschke-Lowenstein disease – a literature review. Chirurgia (Bucur).

[10] Uth Ovesen A (2012). Perianal Buschke-Löwenstein tumour. Ugeskr Laeger.

[11] Orkin BA (2013). Perineal reconstruction with local flaps: technique and results. Tech Coloproctol.

